# Pathogenic role for macrophage migration inhibitory factor in glioblastoma and its targeting with specific inhibitors as novel tailored therapeutic approach

**DOI:** 10.18632/oncotarget.24885

**Published:** 2018-04-03

**Authors:** Katia Mangano, Emanuela Mazzon, Maria Sofia Basile, Roberto Di Marco, Placido Bramanti, Santa Mammana, Maria Cristina Petralia, Paolo Fagone, Ferdinando Nicoletti

**Affiliations:** ^1^ Department of Biomedical and Biotechnological Sciences, University of Catania, Catania, Italy; ^2^ IRCCS Centro Neurolesi “Bonino-Pulejo”, Messina, Italy; ^3^ Department of Medicine and Health Sciences, University of Molise, Campobasso, Italy; ^4^ Department of Formative Processes, University of Catania, Catania, Italy

**Keywords:** macrophage migration Inhibitory factor, glioblastoma, brain tumors, neuro-oncology, D-DT

## Abstract

Macrophage Migration Inhibitory Factor (MIF) is a pro-inflammatory cytokine expressed by a variety of cell types. Although MIF has been primarily studied for its role in the pathogenesis of autoimmune diseases, it has also been shown to promote tumorigenesis and it is over expressed in various malignant tumors. MIF is able to induce angiogenesis, cell cycle progression, and to block apoptosis. As tailored therapeutic approaches for the inhibition of endogenous MIF are being developed, it is important to evaluate the role of MIF in individual neoplastic conditions that may benefit from specific MIF inhibitors. Along with this line, in this paper, we have reviewed the evidence of the involvement of MIF in the etiopathogenesis and progression of glioblastoma and the preclinical data suggesting the possible use of specific MIF inhibition as a potential novel therapeutic strategy for brain tumors.

## MIF, 50 YEARS OLD AND STILL LOOKING (VERY) ATTRACTIVE

Macrophage Migration Inhibitory Factor (MIF) was discovered in 1966 as a molecule capable of inhibiting the migration of macrophages [1], a role that inspired its name. MIF is expressed by several cells such as epithelial, endothelial and immune cells [2]. MIF shares characteristics of cytokine, enzyme, endocrine molecule and chaperon-like protein. MIF binds to its receptor CD74 that provides the binding site, while the downstream signal transduction pathways [MAPK and AKT pathways] are activated via CD44 [3]. Simultaneously, MIF also activates the chemokine receptors, CXCR2 and CXCR4 [4]. Recent studies have revealed that another cytokine named D-DT or MIF-2, that is produced by a gene adjacent to MIF, exhibits biological properties very similar to MIF. Although the sequence homology of D-DT with MIF is only 34%, the overall structural features exhibit strong similarity. Both homologs possess enzymatically binding pockets with a catalytic proline residue, that mediate the tautomerization of the D-dopachrome and p-hydoxyphenylpyruvate (HPP) substrates. Two different end products are produced from D-dopachrome, i.e. 5,6-dihyroxyindole from D-DT and 5,6-dihydroxyindole carboxylic acid from MIF.

From a pharmacological and clinical point, the overlapping biological properties of MIF and D-DT anticipates potential synergisms of these two cytokines in modulation of physiological and pathological effects, that could benefit from simultaneous dual inhibition [5–7].

MIF exerts pleiotropic biological actions that include glucocorticoid antagonism [8, 9], upregulation of Toll-like receptor 4 (TLR-4) expression [10], control of transcriptional effects of JAB1 [11] and suppression of activation-induced, p53-dependent apoptosis, by its direct interaction with p53, and stabilization of the p53-MDM2 complex [12].

**Figure 1:**
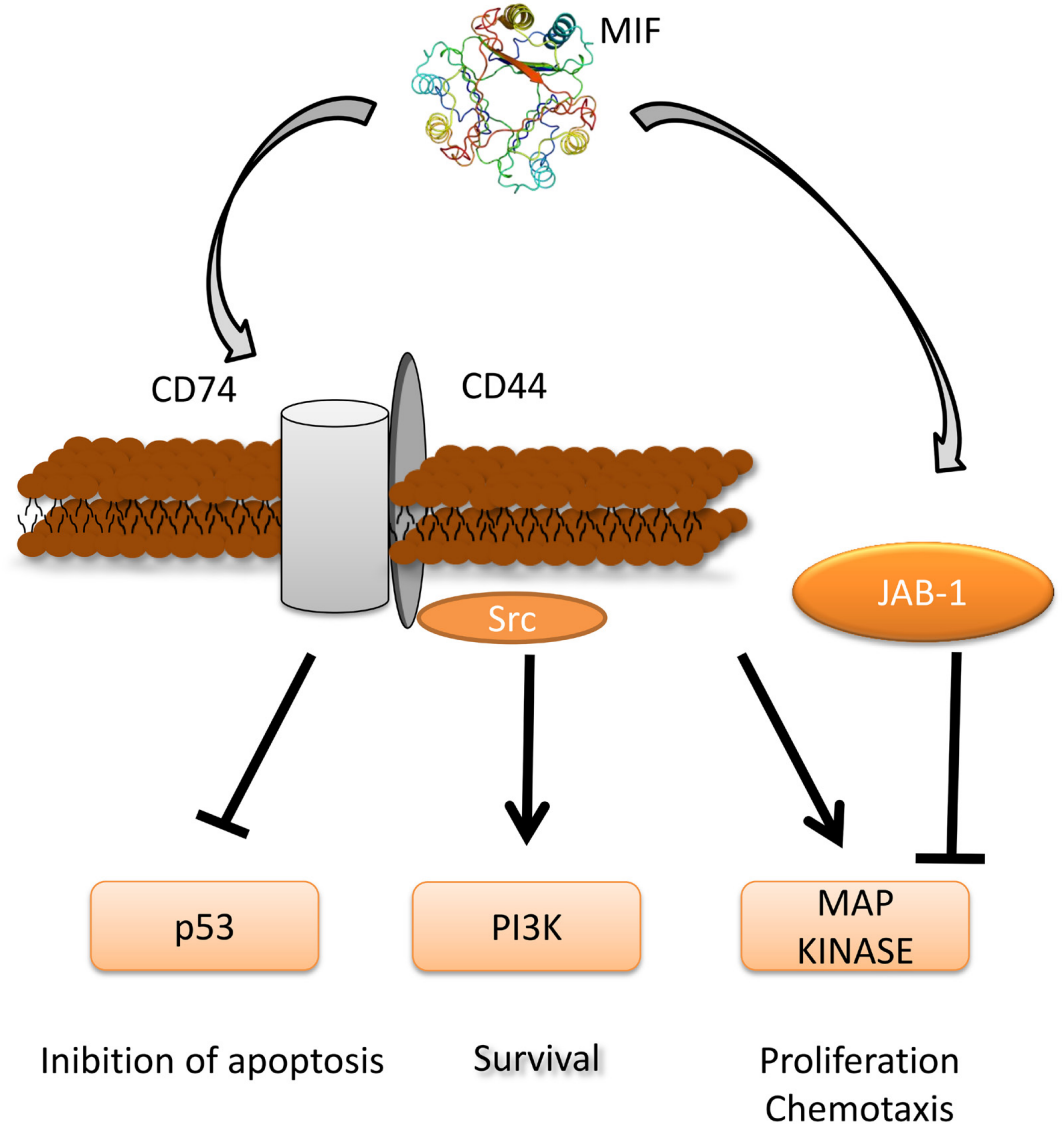
MIF signaling pathway and its role in tumorigenesis Three-dimensional structural data for MIF has been obtained from the Protein Data Bank (https://www.rcsb.org/).

This latter action may sustain inflammatory responses in spite of activation-induced apoptosis, and it may mediate MIF broad inflammatory and proliferative effects on diverse cell types. MIF also promotes the phosphorylation of the ERK1 and ERK2 MAP kinases [13], activates the ERK effectors, cytoplasmic phospholipase A2, which initiates arachidonic metabolism and has a role in p53 suppression [14], and the Elk-1 and Ets transcription factors, which regulate TLR4 expression [10]. MIF-dependent ERK activation also promotes maximal expression of cyclin D1, RB phosphorylation, and adhesion and/or growth factor stimulation of mesenchymal cells [15, 16]. MIF may also contribute to cell proliferation, via activation of the PI3K-Akt signalling pathway [17–19] (Figure 1).

MIF has originally attracted much attention as a central mediator of several immunoinflammatory and autoimmune diseases [20–26]. It has been demonstrated that the expression of MIF is driven by a functional polymorphism that can be observed in about half of the individuals, who carry the 6- 7- and 8-CATT repeat alleles. These individuals produce larger amounts of MIF than those with the 5-CATT repeat allele. Increased production of MIF has been linked to a more aggressive course of immunoinflammatory diseases, such as, asthma and rheumatoid arthritis [7, 27].

## MIF AND CANCER

More recently, the capacity of MIF to upregulate essential steps of tumorigenesis such as angiogenesis [28], cell proliferation, and tumor invasion [28] (Figure 1) and the pharmacological reversibility of these actions by specific MIF inhibitors, [29] have suggested a role of MIF in tumorigenesis and as a chemotherapeutic target. This concept has been reinforced by the observations that MIF may favor the generation of an oncogenic environment by favoring the escape of tumors from immune surveillance, via induction of myeloid-derived suppressor cells in the tumor microenvironment [30], inhibition of T lymphocyte activation [31] and polarization of macrophages to an M1 phenotype [32]. MIF also inhibits the lysing of tumor cells by natural killer (NK) cells [33].

In agreement with these oncogenic properties, both experimental and clinical studies have shown that high levels of MIF are found in several types of human cancers and are apparently implicated in all stages of development of the tumors [17, 34, 35] (Table 1). Upregulated MIF expression has been reported in gastric cancer, pancreatic cancer, melanoma, hepatocellular carcinoma, malignant glioma and cervical adenocarcinoma [28]. In addition, it has been shown that in melanoma, IFN-gamma increases cell surface expression of CD74, and the interaction with its ligand MIF activates the PI3K/AKT pathway, promoting tumour survival [36].

**Table 1:** MIF overexpression in human cancer

MIF overexpression	Comments	Ref
Childhood rhabdomyosarcoma	Human RMS cells express and secrete MIF	[137]
Melanoma	MIF levels increase in advanced stages	[18]
Uveal melanoma	MIF prevents NK cell-mediated lysis	[138]
Gastric cancer	Serum MIF levels increase in advanced stages	[44]
Pancreatic ductal adenocarcinoma	Higher MIF levels correlate with poor prognosis	[42]
Hepatocellular carcinoma	Serum MIF levels are higher in HCC patients MIF levels are increaed in tumor compared to adjiacent non tumor tissue MIF levels correlate with TNM	[40]
Malignant glioma	MIF levels increase wuth tumor grade MIF counteract NK cell-mediated lysis	[83]
Lung adenocarcinoma	MIF distribution predicts patient prognosis	[38]
Non-small cell lung cancer	Higher MIF levels are found in tumor tissue. Higher expression of MIF correlate with poor prognosis	[39]
Oral cavity squamous cell carcinoma	Higher MIF levels are assocated to worse prognosis	[41]
Head and neck squamous cell carcinoma	Tumor tissue MIF expression and plasma levels correlated with tumor recurrence and metastasis, and overall survival.	[35, 43]
Osteosarcoma	MIF levels are increased in the tissue and serum samples of patients and correlate with tumour size, pulmonary metastasis and survival rate	[139]

The molecular mechanism of action by which MIF overexpression in pancreatic cancers is associated with very poor prognosis and marked cancer aggressiveness has been linked to inhibition by MIF of the orphan nuclear receptor NR3C2, that controls aggressiveness and survival in pancreatic ductal adenocarcinoma (PDAC). Specifically, MIF upregulates miR-301b, which targets NR3C2, suppressing its expression. PDAC tumors expressing high levels of MIF showed elevated levels of miR-301b and reduced levels of NR3C2, that predict poorer survival in PDAC patients. NR3C2 also inhibited epithelial-to-mesenchymal transition and increased the sensitivity to gemcitabine, that is the standard of care treatment for PDAC. Furthermore, in a mouse model of PDAC, deletion of MIF impaired the MIF-mir-301b-NR3C2 signaling axis, ensuing into a reduction of metastasis and prolonged survival [37].

Local expression and/or circulating blood levels of MIF have also been proposed as biomarkers of prognosis and therapeutic response. In fact, in contrast to a paper reporting that low nuclear MIF expression correlated with a worse prognosis in lung adenocarcinoma [38], subsequent studies concordantly have shown that high MIF expression/levels correlates with poor patient survival in several types of cancer including, lung cancer, hepatocellular carcinoma, oral squamous cell carcinoma and metastatic melanoma and head and neck squamous cell carcinoma [18, 39–44].

A meta-analysis indicates an association between any C allele in the MIF -173 G/C promoter polymorphism and an increased risk of cancer, particularly for solid tumors. This association appeared stronger for prostate cancer, specifically. In addition, a correlation has been shown between MIF gene polymorphism with the risk of early-stage cervical cancer and lymphatic metastasis [45, 46].

## D-DT (MIF-2) AND CANCER

The D-dopachrome tautomerase (D-DT) is a member of the MIF protein superfamily [6]. MIF and D-DT are encoded on Chromosome 22q11, in close proximity to matrix metalloprotease 11 (MMP11) and the two theta-class glutathione S-transferases, GSST1 and GSTT2. MIF and D-DT are made of three exons, of similar length, and two introns. Also, both genes have consensus binding sequences for SP-1 and CREB at the promoter. It is likely that MIF and D-DT genes derive from an ancestral duplication event [5, 6, 47]. Similarly to MIF, D-DT binds the CD74 ectodomain, although with an approximately 3-fold higher acid dissociation constant (ka) and a 11-fold higher dissociation rate (kd) as compared to MIF [5, 6, 47]. Stimulation of macrophages with D-DT leads to the activation of ERK1/2, and costimulation with both D-DT and MIF shows additive effects. Differently from MIF, however, D-DT lacks the pseudo(E)LR motif that allows MIF to engage the chemokine receptor, CXCR2 [5, 6, 47]. Interestingly, the D-DT gene lacks the CATT5–8 microsatellite repeat, rs5844572, that controls MIF production and is associated to increased severity of rheumatoid arthritis [47].

D-DT is over-expressed in PDAC tissue and its levels correlate with those of MIF. Moreover, in the pancreatic cell line, PANC-1, knockdown of D-DT and MIF was associated to decreased activation of ERK1/2 and AKT, increased p53 expression and reduced tumor growth *in vitro* and *in vivo*. Interestingly, concurrent knockdown of both D-DT and MIF resulted in enhanced inhibition of ERK1/2 and AKT and cell proliferation as compared to single MIF or D-DT shRNA treatment. Also, treatment of PANC-1 cells with the dual covalent tautomerase inhibitor of both MIF and DDT, 4-iodo-6-phenylpyrimidine (4-IPP), reduced proliferation and colony formation *in vitro* and tumor growth in the mouse xenograft model [48]. This is in line with data on the squamous carcinoma cell line, SCCVII [29], and on the A549 lung adenocarcinoma cells [49], where 4-IPP treatment reduced proliferation and invasiveness. MIF and D-DT have also been shown to promote the expression of VEGF and CXCL8 and to antagonize AMPK activation in a CD74-dependent manner in non-small cell lung cancer [50, 51]. In the melanoma cancer cell line, B16F10, siRNA inhibition of D-DT reduced cell proliferation and promoted apoptosis, and *in vivo* blockade of D-DT with anti-D-DT antibodies reduced tumor progression in the xenograft model [52]. A role for D-DT has been also suggested in colorectal cancer, for its ability to regulate the stability and transcriptional activity of β-catenin, partly dependent on COX-2 expression. Indeed, β-catenin expression is significantly decreased in D-DT-deficient cells and it is restored by adenoviral re-introduction of COX-2 [53]. Clear cell renal cell carcinomas have positive staining for D-DT, with 66% of the samples showing moderate-high levels. In addition, D-DT knockdown in RCC4 cells significantly reduced cell survival and growth [54]. D-DT shRNA treatment was associated to more pronounced effects than MIF knockdown and an additive effect could be observed upon dual D-DT and MIF knockdown [54].

These findings suggest that D-DT may vicariate MIF when MIF is pharmacologically suppressed and may explain some of the partial success sometime achieved by traditional single inhibitors of MIF. Accordingly, dual inhibitors capable of simultaneously binding both homologs may warrant studies as novel anticancer drugs.

## THE ROLE OF MIF IN GLIOBLASTOMA

**Figure 2:**
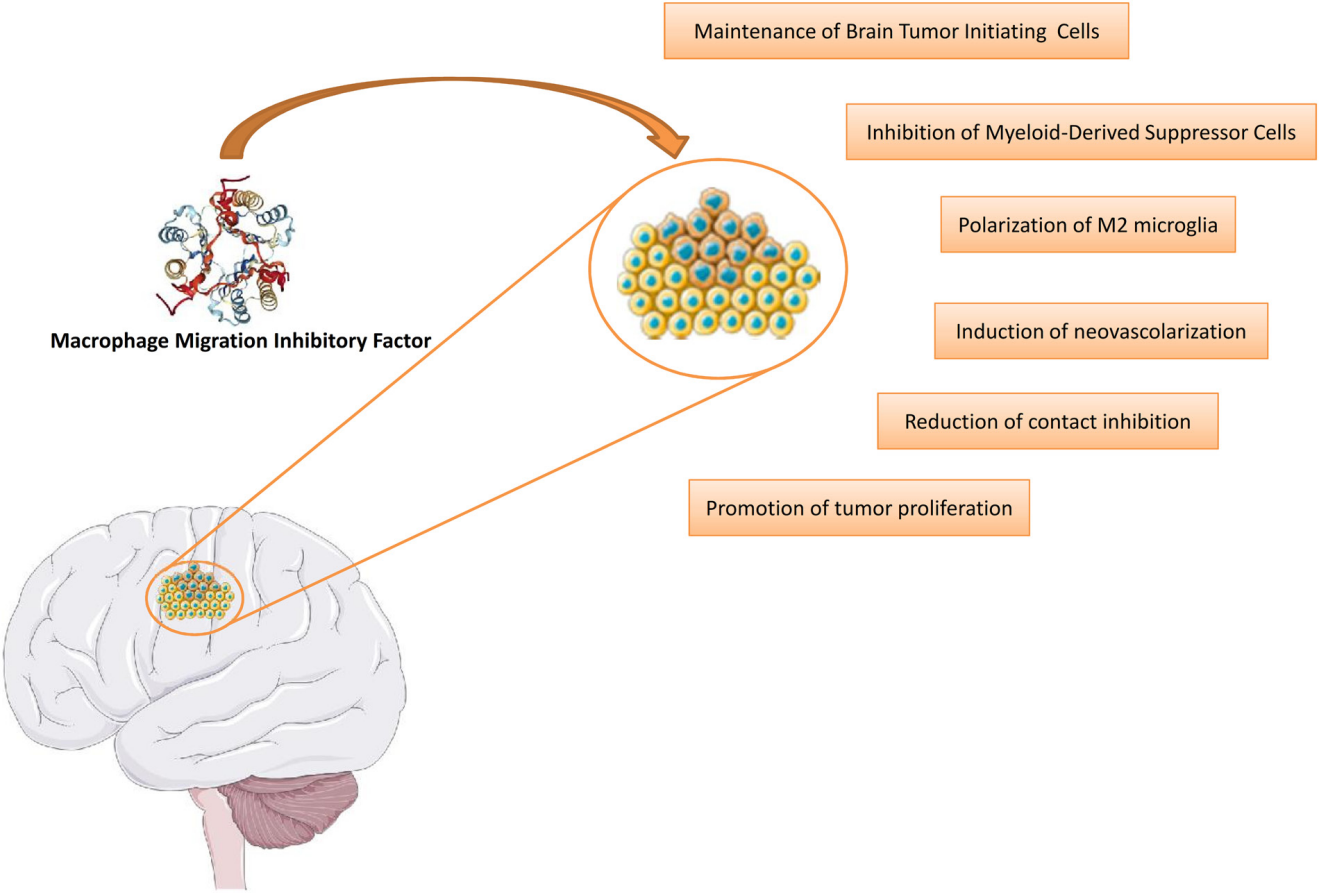
Involvement of MIF in the etiopathogenesis of glioblastoma This figure was drawn using the vector image bank of Servier Medical Art (http://smart.servier.com/). Servier Medical Art by Servier is licensed under a Creative Commons Attribution 3.0 Unported License. (https://creativecommons.org/licenses/by/3.0/). Three-dimensional structural data for MIF has been obtained from the Protein Data Bank (https://www.rcsb.org/).

The increasing evidence supporting a role for MIF in cancer has also attracted attention on the contribution of this cytokine to the pathogenesis of glioblastoma (Figure 2) and the possible development of anti-MIF tailored treatment for this disease.

### Glioblastoma: state of the art, current therapies, unmet medical needs

Glioblastomas, usually located in the cranial hemispheres in the frontotemporal region, are the most common primary tumors in the brain and they are characterized by an aggressive course and poor prognosis and high likelihood of recurrence [55] Genetic and environmental factors contribute to the pathogenesis of gliomas with ionizing radiation representing the highest risk factor. Cerebral irradiation, even at low doses, may increase the incidence of brain tumors with a latency period of 10 to more than 20 years after exposure [56]. As we will discuss more in detail below, currently available strategies for the treatment of glioblastomas are based on open surgery, chemotherapy (temozolomide) and radiotherapy.

According to the 2007 World Health Organization (WHO) classification of tumors of the central nervous system (CNS), glioblastoma (GBM) is defined as a grade IV astrocytoma [57], that is an intrinsic brain tumor developing from glial cells, whose cells are similar to astrocytes [58]. Tumors of grade IV, which are cytologically malignant, mitotically active and tending to necrosis, are usually correlated with fast pre- and postsurgical disease progression and with a deadly outcome [57]. Although GBM primarily affects adults, it can rarely arise as a congenital neoplasm, accounting for about 3–14% of congenital brain tumors [59].

The 2016 WHO report on the CNS defines different tumors considering not only histology but also molecular features, such as the presence of genetic mutations in the Isocitrate dehydrogenase (IDH) 1 and 2 genes [60]. Accordingly, GBMs could be classified into three groups: IDH-wild type (about 90% of cases), IDH-mutant (nearly 10% of cases), and NOS (not otherwise specified, all the cases for which a complete IDH assessment cannot be executed) [61]. IDH-wild type prevails in older patients (median age at diagnosis 62 years) and corresponds to primary or de novo glioblastoma, whereas IDH-mutant mainly affects younger patients (median age at diagnosis 44 years) and coincides with secondary glioblastoma [61]. While primary GBM develops de novo and it is characterized by the lack of a detectable precursor tumor, secondary GBM arises from the malignant evolution of a lower grade glioma, such as an grade II or grade III astrocytoma [62].

GBM, which is the most malignant glioma, is characterized by necrosis, vascular proliferation and histological heterogeneity [63] and represents the most frequent glial tumor, with an incidence of nearly 3/100,000 cases per year [64]. By performing a meta-analysis of preceding genome-wide association studies (GWAS) and two new GWAS, Melin et al have found 13 new glioma risk loci (5 for GBM and 8 for non-GBM tumors) [65]. These results confirm that there are multiple differences in the genetic susceptibility to GBM and non-GBM tumors, thereby suggesting that glioma subtypes could have distinct etiologies [65].

Currently, the GBM standard of care consists of maximal safe surgical resection, followed by radiotherapy and chemotherapy with temozolomide [66]. If gross total resection is not feasible, a diagnostic biopsy for histological and molecular analyses needs to be performed [60]. Although surgery is fundamental to treat GBM, it is not sufficient due to the axonal spreading of GBM cells [60]. For more than 30 years, postsurgical radiotherapy has been considered the standard treatment for new cases of GBM [67] resulting in increased survival of 6 months [60]. The standard protocol of radiotherapy consists of a dose of 50–60 Gy in 1.8–2.0 Gy fractions to be delivered to the tumor volume with a finite margin of about 2-to-3-cm for the planning target volume [60]. The addition of the oral alkylating agent temozolomide, to radiotherapy allows a significant, although still marginal benefit, increasing median survival from 12.1 to 14.6 months [55]. In addition, there is a subgroup of GBM patients poorly responsive to temozolomide [67] who are those with unmethylation methyl-guanine methyl transferase (MGMT) gene promoter in the tumor tissue [67]. Moreover, overall survival may be considerably increased by the addition of tumor-treating fields which are antimitotic treatments that use alternating electric fields to interfere with cell division, to adjuvant temozolomide chemotherapy [68]. In case of tumor progression, treatment with bevacizumab against circulating vascular endothelial growth factor (VEGF), sometimes associated with lomustine (CCNU), is adopted [69]. Glucocorticoids (GCs) are also frequently used in GBM, since they diminish the edema associated with the tumor [70]. In addition, GCs may induce MAPK phosphatase 1 (MKP-1), thereby inhibiting the migration and invasion of GBM cells [70].

In spite of these multiple therapeutic options, the prognosis for GBM patients remains dramatically poor and makes the disease a clear unmet medical need with a median survival period of 14,6 months and less than 5% of patients surviving for 5 years [66]. The major obstacles in the treatment of GBM are the difficult penetration of the drugs through the blood–brain barrier (BBB), the inter- and intra-tumoral genetic heterogeneity, along with the tumor ability to induce immunosuppression in the surrounding microenvironment, and its recurrence [71]. A further relevant issue is the presence of cancer stem cells (CSCs), which are self-renewing and tumorigenic cells that could modulate the immune system and cause therapeutic resistance and tumor recurrence [72, 73].

### Novel approaches for the treatment of GBM

In the recent years, considerable progress has been made in the field of immunotherapy and numerous preclinical and clinical data about immunotherapy strategies for GBM have arisen [71, 74]. Immunotherapeutic approaches include: vaccines which could be classified, according to their sensitization target, in: whole tumor vaccines, tumor-associated antigens and tumor-specific antigens; cell-based therapies, which consist of the administration to the patients of effector immune cells, already sensitized and activated against tumor targets; and immune checkpoint modulators, which consist of antibodies blocking inhibitory immune checkpoint molecules, such as PD-1 and PDL1 [74]. However, the principal reason of the limited success of chemotherapeutic drugs against gliomas is the inability to overcome the blood–brain barrier (BBB) and to reach the tumor tissue. Several efforts have been made to enhance drug delivery to the brain, including: tight junction opening, via infusion of hyperosmotic agents; surfactants or bioactive molecules; chemical modification of the drug, in order to create more lipophilic prodrug; inhibition of efflux transporters present at the BBB, such as P-gp; convention-enhanced drug delivery; and craniotomy-based drug delivery [75]. Another recent and promising therapeutic strategy is based on nanotechnology [72, 76–78]. Colloidal nanocarriers, liposomes, polymeric nanoparticles (PNPs) and lipid nanocapsules (LNCs) could ameliorate effectiveness, diminish non-specific toxicity and raise stability of drugs, and could also facilitate drug delivery to the brain tumors, which is hampered by the BBB [75]. The principal features for brain-targeted nanocarriers are represented by the size and their surface charge, as well as the presence of hydrophilic polymers and ligands on the surface. Hence, cationic nanocarriers with hydrophilic surface coating are most suitable for targeting drugs to the brain [75]. Liposomes have been largely tested in gliomas, both in the preclinical and in the clinical setting, showing improved drug accumulation within tumor. As for PNPs, Poly(butyl cyanoacrylate) (PBCA) NPs, with surface coating of polysorbate 80, loaded with doxorubicin have been tested by independent groups in different GBM models [75]. Finally, LNCs represents hybrids between liposomes and polymeric nanocapsules with a liquid core surrounded by a shell of solid lipid molecules. They allow the preferential accumulation of drugs in brain tumors, and promising preclinical data are available [75]. Although several *in vitro* and *in vivo* studies have been carried out to evaluate the efficacy of these nanocarriers in the treatment of GBM, only liposomes have reached phase I/II clinical trials [75]. Since the intratumoral genetic heterogeneity (ITH) of GBM is an important cause of the poor prognosis, the development of new technologies (CRISPR-Cas9 screening, CyTOF, cellular barcoding, single cell analysis), that can identify GBM ITH and cells resistant to treatment, could open new therapeutic windows [79].

### Can MIF play a key pathogenetic role and represent a therapeutic target in GBM?

Increasing body of work indicates that MIF plays an important pathogenetic role in malignant progression of GBM and other CNS tumours [80, 81]. In particular, a strong increase of MIF expression in human GBM has been reported [62, 63].

In GBM tissues, MIF localizes particularly in close proximity of necrotic areas and in tumor cells surrounding blood vessels and its expression is frequently associated with the presence of the tumor-suppressor gene p53. Another study has shown that expression of the MIF receptor CD74 in GBM may be involved in the resistance to temozolomide [63, 82]. The analysis of the *in vivo* levels of MIF expression in 166 gliomas and 23 normal control brains by immunohistochemistry has shown that MIF immunoreactivity was increased in WHO grade II gliomas and increased significantly in higher tumour grades (III-IV) [83]. Also, MIF transcripts were elevated up to 800-fold in malignant glioma cells compared with normal brain. This correlated to high protein levels in total cell lysates and of secreted MIF. Wild-type p53-retaining glioma cell lines exhibited higher levels of MIF, which is in line with the observation that MIF may act as a negative regulator of p53 signalling in tumour cells [83].

An association has also been reported between elevated expression of MIF and tumor recurrence and poor prognosis of patients with gliomas [84].

In agreement with these data, strong MIF expression has been observed in primary glioblastoma cells [80]. In addition, the MIF inhibitor ISO-1 inhibited the proliferation of glial cells in a concentration-dependent manner [80, 85]. Furthermore, hypoxia, as cell stressor, increases the protein expression of MIF in primary GBM cells [85]. Another *in vitro* study has shown that specific blockade of MIF in GBM cells reduced the growth rates of tumor cells, both under confluent and over-confluent conditions, thus anticipating a role of MIF in overcoming contact inhibition. Several proteins involved in contact inhibition including p27, p21, p53 and CEBP alpha were upregulated when endogenous MIF was blocked *in vitro*, indicating a restoration of contact inhibition in the tumor cells [86]. The authors have also shown that inhibiting MIF intrinsic tautomerase activity by the small compound inhibitor ISO-1, reduced proliferation and mitogenic signaling in glioblastoma cells [86].

In glioma stem cells (GSC), it has been shown that miR-608 negatively regulated MIF expression by direct targeting its 3'UTR. miR-608 overexpression significantly reduced the proliferation, migration and invasion, and promoted apoptosis of GSCs by downregulating MIF [87].

MIF expression in human GBM has also been shown to correlate with that of vascular endothelial growth factor and with angiogenesis [88]. In particular, it has been demonstrated that upregulation of MIF in glial tumour cells is induced by hypoxic and hypoglycaemic stress [63] and that MIF and CXCR4 colocalize in hypoxic area in glioma specimens [89]. *In vitro*, exposure of the glioblastoma cells lines, U87 and U251, to hypoxia was associated to an increase in MIF and CXCR4 levels and to the induction of vasculogenic mimicry [89]. Neutralization of MIF or administration of the a CXCR4 antagonist, AMD3100, or the PI3K inhibitor, LY294002, significantly inhibited vasculogenic mimicry formation and epithelial mesenchymal transition [89]. Furthermore, hypoxia-induced MIF expression is regulated by HIF-1alpha, via a HRE in the 5'UTR of the MIF gene, and it is further augmented by CREB [90]. Indeed, over-expression of HIF-1α induces MIF expression, which is blocked by mutation of the HRE in the 5′UTR. Moreover, over-expression of CREB blocks hypoxia-induced MIF promoter activity [90]. These data suggest that hypoxia-induced MIF expression is regulated by HIF-1α but increased by hypoxia-induced degradation of CREB.

That MIF may be associated with angiogenesis in GBM is also consistent with the demonstration that the high levels of MIF (along with other cytokines) in GBM significantly decline after 1 day of treatment with the antiangiogenic drug aflibercept [91].

Nonetheless, the role of MIF as angiogenetic factor in GBM has recently been questioned by the evidence that bevacizumab resistance in GBM is driven by reduced MIF at the tumor edge causing proliferative expansion of M2 macrophages, which in turn promotes tumor growth [92]. Hence, MIF might have both pro-tumorigenic and anti-tumorigenic effects, depending on determined circumstances: first, the cells by which it is produced, stromal versus tumour ones; second, the microenvironment and the cytokine milieu; third the effects might be related to the dose, being protumoral either at excessively low or high levels; fourth, post-translational modifications, such as glycosylation or N-cysteinylation, partially influenced by ROS levels [92].

However, with this caveat in mind, the current evidence ultimately supports a proangiogenic and oncogenic role for MIF in angiogenesis in GBM.

### MIF as an immune checkpoint inhibitor in GBM

In light of the clear clinical efficacy demonstrated by immunomodulatory approaches in the treatment of several types of cancers [93], much attention has recently been focused on the possible role of MIF as an additional immune check-point inhibitor, capable of generating an oncogenic environment at the tumor site during GBM development and maintenance. Most of the studies in GBM actually indicate that local production of MIF might be associated with a milieu favoring tumor escape from immune surveillance and its action seems to be primarily, but not exclusively exerted, at the level of microglia cells and regulation of their functions [94].

Histopathological and flow cytometry studies of human and rodent gliomas have demonstrated the heterogeneity of the tumor and its niche, that is primarily composed of reactive astrocytes, endothelial cells, and numerous immune cells. The number of glioma-associated microglia/macrophages (GAMs) and MDSCs is the highest in gliomas and inversely correlates with patient survival. Although GAMs maintain some functions of innate immune cells, their ability to mount an efficient anti-tumour response via TLRs, cytokines, and upregulation of co-stimulatory molecules is impaired. Moreover, tumor-reprogrammed GAMs secrete immunosuppressive cytokines and chemokines that downregulate antitumor responses. Both GAMs and MDSCs can attract regulatory T lymphocytes to the tumor, but MDSCs inhibit immune-mediated cytotoxic responses [95, 96].

It has also been reported that microglial cells in the brain tumor microenvironment persist in a M2 phenotype at the peritumoral site, promote the growth of gliomas and are associated with enhanced glioma malignancy. The possible contribution of MIF to the persistent M2 oncogenic phenotype of microglia in GBM has also been studied [32]. It has been shown that brain tumors escape pro-inflammatory M1 conversion of microglia via CD74 activation through the secretion of MIF which results in a M2 shift of microglial cells. Inhibition of this glioma-microglial interaction through anti-MIF antibody or small interfering RNA (siRNA) treatment exerts beneficial effects in preclinical models by reinstating the microglial pro-inflammatory M1 function [32]. In particular, inhibition of (IFN)-γ secretion in microglia seems to play a crucial role in the prooncogenic role of MIF, as blockade of MIF/CD74 interaction promotes IFN-γ release and amplifies tumor death. The reinstated IFN-γ secretion leads both to direct inhibition of glioma growth as well as inducing a M2 to M1 shift in glioma-associated microglia [32]. Accordingly, interference with the MIF signaling pathway may represent a viable therapeutic option for the restoration of IFN-γ-driven immune surveillance [32].

It has also been demonstrated that mast cells (MCs) infiltrate the brain during GBM and that MIF plays a key role in favouring MC infiltration in GBM. This may be pathogenically important as MCs are key modulators of the tumor microenvironment, influencing angiogenic and immune-environmental processes, as well as tissue remodeling [96, 97].

In particular, the accumulation of MCs, which is dependent on the malignancy grade of the glioma, correlates with the level of MIF expression. In addition, a direct correlation has been reported between the level of pSTAT5 in MCs and the level of MIF [98].

Other Authors have reported that stable knockdown of MIF by shRNA in glioma cells increased tumour cell susceptibility towards NK cell- and CD8+T cell- mediated cytotoxicity by downregulating the immune receptor NKG2D on NK and CD8+ T cells [83].

Otvos and coworkers have identified immune-suppressive myeloid-derived suppressor cells (MDSCs) in GBM patients brains nearby cancer stem cells (CSCs). Depletion of MDSCs by 5-fluorouracil (5-FU) resulted in prolonged survival in a mouse model of glioma. Also patient-derived CSCs specifically promoted MDSC-mediated immune suppression. CSCs secreted multiple factors promoting this activity, including MIF, which was produced at high levels by CSCs. MIF increased the production of arginase-1 in MDSCs, while MIF targeting reduced arginase-1 production. Similarly to 5-FU, targeting tumor-derived MIF prolonged survival to tumor-bearing animals and increased the cytotoxic T cell response within the tumor. Along with the lack of effects of MIF inhibition on the viability of tumour cells, these data indicate that MIF is primarily an indirect promoter of GBM progression, acting through suppression of immune rejection by activating and protecting immune suppressive MDSCs within the GBM tumor microenvironment [73].

In support of the role played by MIF as immune check-point regulator in GBM, *in vitro* treatment with the MIF inhibitor, sulforaphane, suppressed the transformation of normal monocytes to MDSCs by glioma-conditioned media [99].

Along this line of research, it has been reported that MIF enhances autophagy in GBM by regulating ROCK1 activity and that it contributes to the escape of dendritic cell surveillance [81].

However, in contrast to these numerous convergent findings on the detrimental role of MIF in favouring anti-tumour immune response another study has demonstrated that MIF receptor CD74 expression in human gliomas is restricted to microglia/macrophages and positively associated with patient survival [100]. The significance of this finding remains to be reconciled with the other studies and reasons for possible apparent discrepancies remain to be established.

### MIF as driver and maintainer of a brain tumour initiating cells

Understanding the pathways that regulate differentiation, growth and maintenance of brain tumour initiating cells (BTIC) is important for the better understanding of pathogenic mechanisms operating in GBM and to design tailored therapeutic approaches.

Fukaya has first described a role for MIF in maintaining the tumorigenic capacity of BTIC, including GBM, by direct inhibition of p53 activity. MIF expression in BTICs was higher than in non-BTICs and human astrocytes. In tumor-derived neurosphere culture *in vitro*, BTICs cultured from GBM patient tumors were expanded longer than non-BTICs. MIF gene knockdown in BTICs resulted in both reduced cell proliferation and increased apoptosis *in vitro*. In a human BTIC mouse xenograft models, MIF gene silencing ameliorated the course of the disease. The study also reported that intracellular localization of MIF in glioma cells and its binding to p53 [101].

These data fit in with previous studies suggesting a mechanistic mode of action by which MIF maintains the tumorigenic capacity of BTIC through an up-regulatory action on Chromatin Helicase-DNA-binding protein 7 (CHD7) [5]. It is of interest that the expression of CHD7, along with that of CHD1, CHD4 and CHD9 genes, is upregulated in GBM, in contrast to that of CHD3 and CHD5 genes that is downregulated. Ongoing work, from this group [102] aims at identifying the mechanisms of MIF signalling, focusing on BTIC epigenomics. Of note, MIF expressed in human induced Pluripotent Stem cell (iPCS), regulates cell proliferation, suggesting a role for MIF in promoting cell proliferation of many stem cell types, including NSPCs, BTICs, and iPSCs [102, 103].

## THERAPEUTIC PERSPECTIVES

The large body of data accumulated on the role of MIF in oncogenesis suggests that MIF may represent a therapeutic target in several cancers including GBM [104].

A summary of MIF antagonists as chemotherapeutic agents is presented in Table 2.

**Table 2:** Strategies for MIF Inhibition in the Treatment of Cancer

Drugs	*In vitro* studies	*In vivo* studies	Clinical trials	Ref
**Small molecule disruption of MIF biological activity**				
Binding in the active site (competitive inhibition);				
– ISO-1	x	x		[80, 85, 86]
– ISO-66	x	x		[116]
– CPSI-2705 and CPSI-1306	x	x		[117]
– SCD-19	x	x		[118]
– Debio1036	x	x		[140]
Covalent linkage to Pro1 (irreversible inhibition);				
– 2-oxo-4-phenyl-3-butanoate	x			[120]
– 4-IPP	x			[49]
– acetaminophen analogs	x	x		[121]
– epicatechins	x			[122]
Allosteric inhibition;				
– ebselen	x			[105]
– ibudilast	x			[106]
– p425	x			[107]
**Indirect destabilization of MIF**				
– 17AAG	x	x		[125, 126]
**Monoclonal antibodies directed against MIF or its CD74 receptor**				
– BaxG03, BaxB01, and BaxM159	x	x		[128]
– Milatuzumab (anti-CD74)	x	x	x	[129–131]

**Figure 3:**
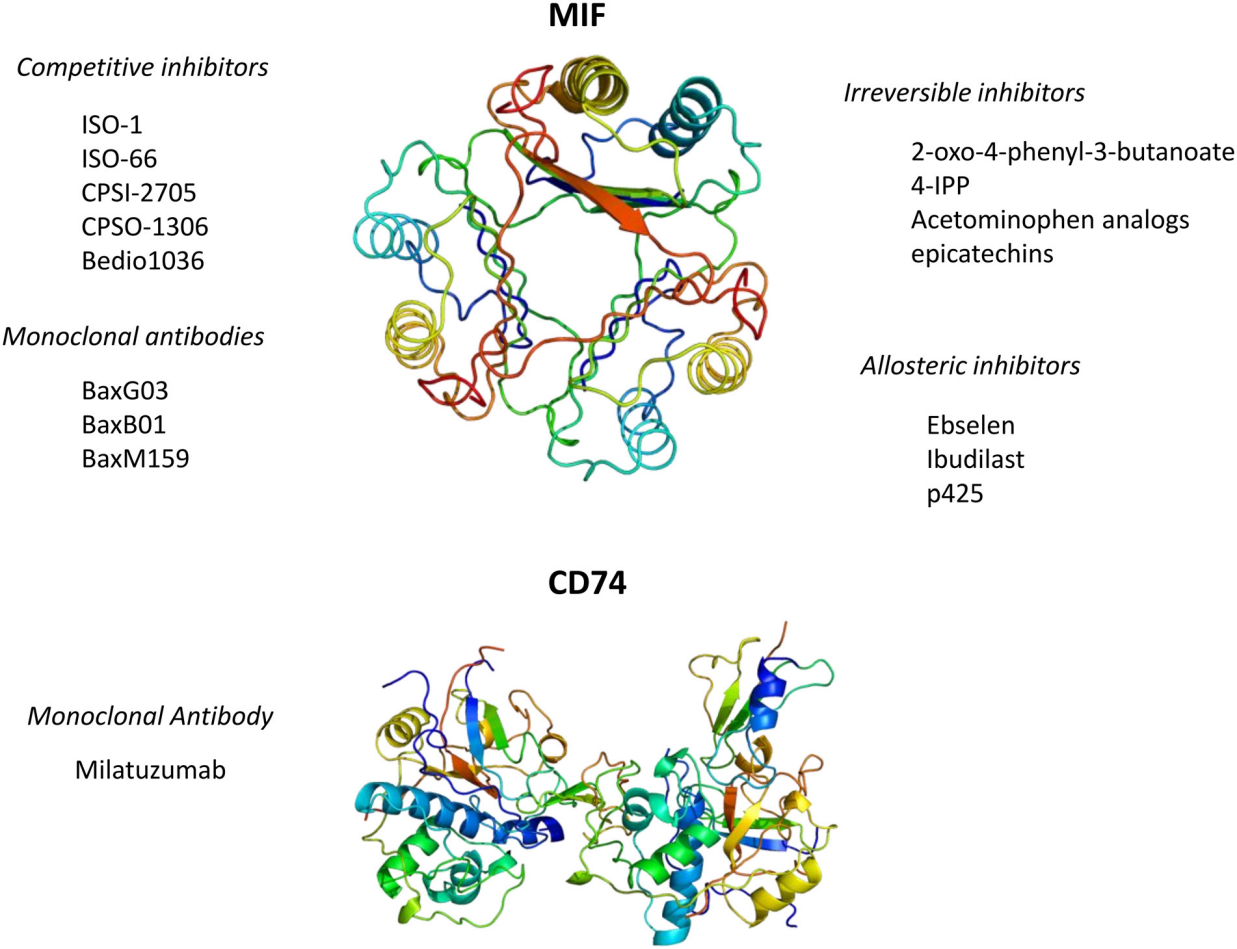
Currently available pharmacological strategies for the inhibition of MIF signaling Three-dimensional structural data for MIF and CD74 have been downloaded from the Protein Data Bank (https://www.rcsb.org/).

The unique and pleiotropic functional characteristics of MIF make possible to identify several approaches by which specific MIF inhibition can be achieved and that include: small molecule disruption of MIF biological activity indirect destabilization of MIF or monoclonal antibodies directed against MIF or its CD74 receptor (Figure 3). A detailed description of these approaches is recently and very accurately reviewed elsewhere [7].

Inhibition of the enzymatic active site of MIF with small molecule competitive inhibitors has also been studied as a strategy to generate MIF inhibitors. The tautomerasic site of MIF has been largely studied with the aim to generate specific MIF-inhibitors. MIF antagonists may interact with the tautomerase active site through different ways [105] including, competitive inhibition, irreversible inhibition, allosteric inhibition, and MIF stabilization, as described below. Competitive inhibition of MIF and covalent linkage of small molecules to Pro1 are the most widely used approaches used to date. Allosteric inhibitors such as, ebselen, ibudilast, and 6’-[(3,3-dimethoxy[1,1’-biphenyl]-4,4’-diyl)bis(azo)]bis[4-amino-5hydroxy-1,3-napthalenedisulphonic acid] tetrasodium salt (p425) have also been described [105–107].

Finally, the possibility to deliver MIF inhibitors using nanoparticle-based drug carriers is currently under development, although the techniques and methodologies may vary [75, 108–111].

### Competitive inhibitors

The isoxazoline class of compounds has been largely studied as prototype of competitive MIF inhibitors. The best studied MIF inhibitor of this class is represented by ISO-1 [S,R-3-(4-hydroxyphenyl)-4,5- dihydro-5-isoxazole acetic acid methyl ester] [105, 112].

MIF-inhibition by ISO-1 has been proven to inhibit viability and function of several human cancer cell lines such as, A549, DU145, LN229, LN-18 and HS683 [49, 70, 113, 114].

*In vivo*, ISO-1 exerts beneficial effects in models of prostate and colorectal cancer [115]. In spite of these promising results, the *in vivo* enzymatic kinetics of ISO-1 and its apparent lack of efficacy, when administered *per os*, has hampered the clinical development of this compound. ISO-66 is an ISO1 derivative, characterized by enhanced stability and lower toxicity than the parental compound ISO-1 [116]. ISO-66 was non toxic and effective in animal models of melanoma and colorectal cancer [116]. *Ex vivo* studies indicated that the effect was secondary to the generation of antitumor-specific effector cells, including recovery of tumor-specific CTL and NK cells tumor killing functions.

Along this line of research, Cytokine PharmaSciences, Inc. has developed two orally available oxazoline derivatives for specific inhibition of MIF, namely CPSI-2705 and CPSI-1306. CPSI-2705 and CPSI-1306 significantly ameliorated the course of the disease in murine bladder and skin cancer models [117]. Another MIF inhibitor, the novel isocoumarin compound, 3-(2-methylphenyl)-isocoumarin, has also recently been described by Mawhinney et al. who demonstrated its ability to ameliorate the course of the disease in a murine model of lung cancer, regardless of whether it was administered upon a prophylactic or therapeutic fashion [118].

Significant evidence has also been generated on the possible regulatory role of endogenous MIF generation and function of regulatory T cells during oncogenesis [119]. Along this line of research, it was shown that the growth rate of CT26 colon carcinoma cells was significantly lower in MIF knockout (MIF(-/-)) mice than in wild-type (MIF(+/+)) mice. This was associated to lower levels of tumor-associated CD4(+)Tregs in MIF (-/-) than MIF(+/+) mice. MIF(-/-) mice also had decreased CD8(+)Tregs and increased CD8-induced tumor cytotoxicity. Interestingly, spleen cells from MIF (-/-) exhibited greater inducible Treg response to anti-CD3/CD28 plus IL-2 plus TGF-β than those from MIF(+/+) mice. Also, spleen cells of (MIF−/−) mice, upon stimulated with anti-CD3/CD28, synthetized less IL-2, but not TGF-β, than those of (MIF+/+) mice. This was reverted by adding recombinant MIF. On the other hand, addition of anti-MIF mAb suppressed anti-CD3-induced IL-2 production by splenocytes of MIF(+/+) mice and downregulated the generation of inducible regulatory T cells. Finally, when exogenously-administered to tumor-bearing MIF(-/-) animals, IL-2 restored the generation of Tregs and tumor growth. Hence, MIF seems to favor tumor growth by increasing Treg generation, through the modulation of IL-2 production. This highlights an intriguing role of endogenous MIF as important inhibitory checkpoint in oncogenesis as tumor-derived CD4+ Tregs play an important prooncogenic role by suppressing the immune response to tumor cells [119].

### Irreversible inhibitors

The terminal nucleophilic proline of MIF makes it an ideal candidate target for covalent inhibitors such as, 2-oxo-4-phenyl-3-butanoate, phenylpyrimidines, acetaminophen analogs, and epicatechins [120–122]. 4-Iodo-6-phenylpyrimidine (4-IPP) has also been identified as a potent inhibitor of the tautomerasic action of MIF activity [49]. 4-IPP acts by irreversibly binding to the Pro1 residue of MIF through nucleophilic displacement of an aromatic iodo group. 4-IPP has an IC50 value that is ten times lower than that of ISO-1. Also, 4-IPP has already been shown to exert powerful anticancer activities in lung cancer cells as well as head and neck squamous cell carcinoma cell line SCCVII *in vitro* [49]. More recently, it has been demonstrated that 4-IPP is the first identified dual D-DT/MIF inhibitor [29].

Dietary isothiocyanates (ITCs), found in cruciferous vegetables, are also MIF inhibitors. The anti-inflammatory and anticancer effects of these compounds have long been recognized, but their mechanism of action remains partly obscure. Recently, it was demonstrated that ITCs potently inhibit MIF tautomerase effects by covalent modification of Pro1 [123].

Although several mechanisms can underlie the anticancer effects of ITCs, their ability to bind and modulate MIF activity may also contribute to this action [124].

### Destabilization of MIF

An alternative way to neutralize the biological activity of MIF has recently been described [125, 126].

MIF is a novel client of HSP90 in cancer cells, and this prevents its degradation. When HSP90 inhibitors are used in cancer cell lines, this leads to augmented degradation of MIF, that is accompanied by acquisition of favorable anticancer activities [125, 126]. It has been shown that the HSP90 inhibitor 17-(alkylamino)-17-(demethoxygeldanamycin) (17AAG) reduced levels of MIF protein and cell proliferation [125]. As augmented levels of HSP90 and MIF are specifically upregulated in cancer cells, tailored inhibitors of HSP90 may represent alternative approaches for MIF inhibition in cancer. Several HSP90 small molecule inhibitors are in clinical trials for cancer [127]. In this regard, it has also been demonstrated that HER2/Erb2 overexpression in breast cancer controls the major oncogenic growth factor HSF1 [126], that also regulates the fate of HSP90 and its clients [126]. Accordingly, inhibition of HER2 suppresses activation of HSP90 with consequential destabilization of MIF [126].

### Monoclonal antibodies against MIF or its receptor CD74

The application of anti-MIF monoclonal antibodies in cancer has only recently been explored. The anti-MIF monoclonal antibodies BaxG03, BaxB01, and BaxM159 have been developed at Baxter (now Shire) that have shown potent dose-dependent *in vitro* and *in vivo* chemotherapeutic effects in human PC3 prostate cancer cells [128]. Similarly, anti-MIF monoclonal antibodies ameliorated the course of the disease in a CT26 colon cancer model [128]. A subsequent Phase 1 trial has been initiated using anti-MIF monoclonal antibodies to treat patients with solid tumors (clinicaltrials.gov, NCT01765790).

Targeting the CD74 receptor to block both MIF and D-DT/MIF2 activity is also a possible strategy to block the action of these cytokines. The anti-CD74 humanized monoclonal antibody, milatuzumab has been shown to synergize with other chemotherapeutic agents and elicits significant antitumor effects in mice [129].

Phase I and I/II studies have been run with preliminary satisfactory results with this antibody in patients with previously treated B-cell lymphomas [130, 131].

Taken as a whole, these data with specific MIF-inhibitors strongly support a pivotal and pleiotropic role of MIF in oncogenesis and also indicate it as a potential chemotherapeutic agent and additional immune check point inhibitor.

### Other MIF inhibitors

Other interesting perspectives have emerged searching for endogenous MIF inhibitors, such as vitamin E, which is capable of binding the active site and to alleviate not only enzymatic activity, but also pro-inflammatory cytokines production [132]; thyroxine, which might fit in MIF hydrophobic pocket, reducing inflammatory effects [133]; and NM23-H1, which physically interacts with the cytokine, through cysteine residues, attenuating MIF-induced p53 suppression [12].

Recent studies by Bloom et al. have found that the anti-rheumatic drug, iguratimod, inhibits MIF *in vitro* and *in vivo* and also synergizes with glucocorticoids. These data are of particular interest, as iguratimod is already used in the clinical setting and could be repurposed for neoplastic conditions characterized by upregulated MIF activity [134].

## *IN VITRO* AND *IN VIVO* EFFECTS OF MIF INHIBITION IN GBM

Monoclonal anti-MIF antibodies have been tested in experimental models of GBM and have provided significant results, especially *in vitro* where they were able to considerably reduce the growth of LN18 and LN229 glioma cells with maximal results under confluent culture conditions [86]. Moreover, these antibodies successfully reduced, in a dose-dependent manner, the migration of MC cells, due to MIF blockade in the other glioma cell line U-2987 MG [98] and diminish the CXCR2 mediated arginase-1 production, an immunosuppressive enzyme, in myeloid derived suppressor cells [73]. However, more studies, especially *in vivo*, are needed to confirm the potential clinical efficacy of anti-MIF monoclonal antibodies in GBM.

The MIF inhibitor ISO-1 inhibited G8 and G9 glioblastoma cells proliferation at 25-50 μM and also led to an up-regulation of MIF protein and its receptors expression, probably as a compensatory feedback of MIF function inhibition [85]. Furthermore, this compound has stronger effects on the LN18 cells than the monoclonal antibodies, not only blocking extracellular MIF but also the intracellular protein [86]. In addition, while GCs alone did not suppress Hs683 glioma cells migration and invasion, the combined treatment with ISO-1, at the dose of 1000 μM, and 1μM dexamethasone markedly reduced both parameters, suggesting that MIF blockade can be a way to sensitize glioma cells to GCs effects [70].

Besides small molecules compounds, antisense plasmids has been tested for their ability to antagonize MIF in experimental GBM. MIF targeting by antisense transfection diminished LN18 glioblastoma cells growth and re-established contact inhibition through the up-regulation of various proteins, such as p27, p21, p53 and CEBP alpha [86]. It has also been discovered that miR-608 that binds MIF 3'UTR, reduces its expression and attenuates U87 and U251 cells proliferation, migration and invasion, inducing apoptosis, due to the lack of survival signaling, as a consequence of the down-regulation of PI3K/AKT and JNK pathways [87]. As far as shRNA action is concerned, it has been noted that it is able to extend tumor latency in animals after intracranial injection of GL261 cells and to induce augmentation of CD8-positive CTL and reduction of Treg lymphocyte in their brain [73]. Moreover, it has successfully resulted in the increase of LNT-229 cells susceptibility to NK killing, thanks to the restoration of NKG2D activating signal [83].

In addition, CD74 shRNA increased U87 cells response to temozolomide [82] and blockade of CXCR4, through the small molecule antagonist AMD-3100 diminished intracranial growth and proliferation of the tumor and augmented apoptosis, due to the decreased activation of ERK-1 and 2 and AKT [135].

**Figure 4:**
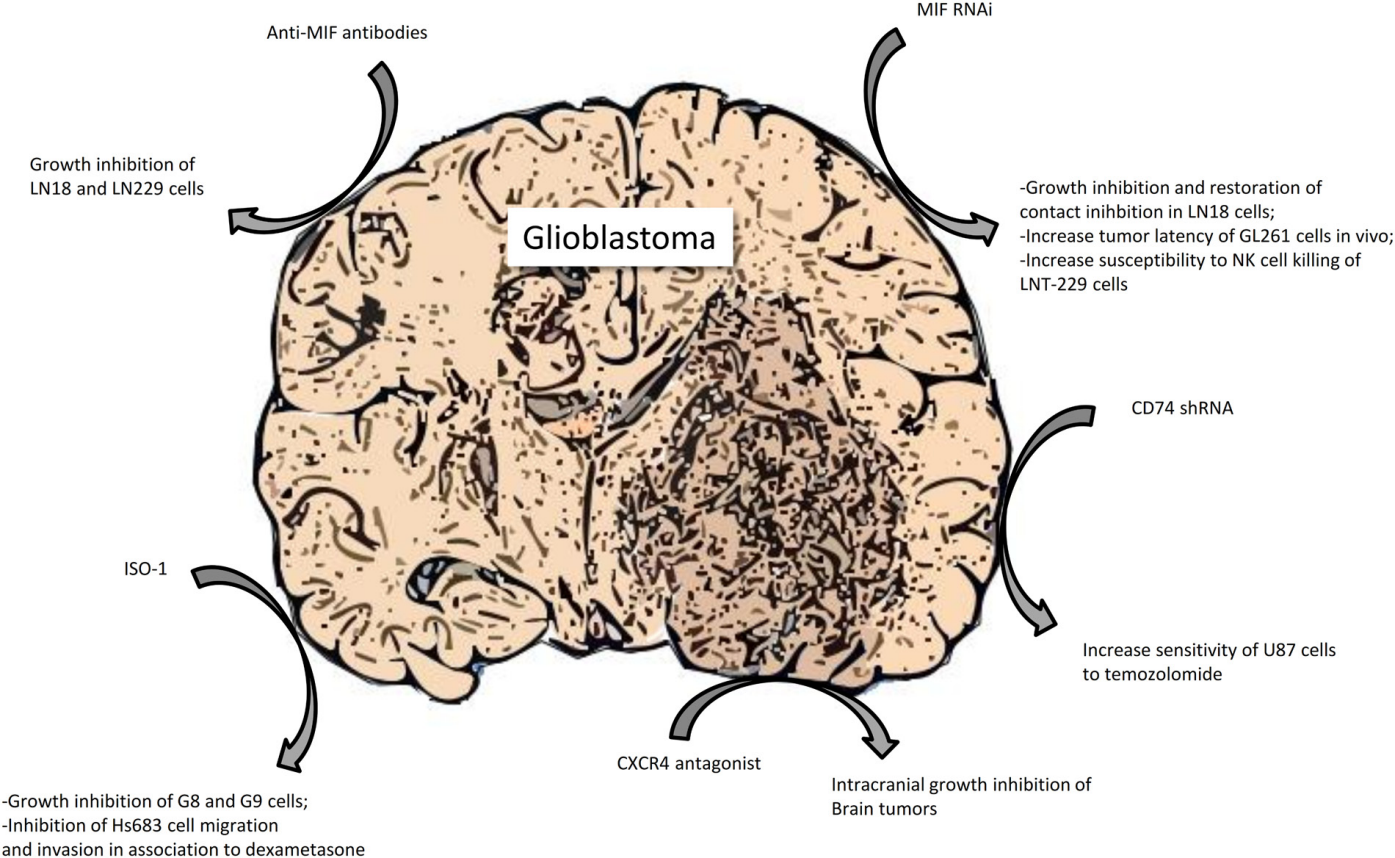
Preclinical evidence of a therapeutic role for MIF signaling inhibition in glioblastoma

It has also been found that anti-angiogenic therapy with bevacizumab is able to induce the depletion of MIF in glioblastoma cells [92]. Two mechanisms have been recognized: suppression of VEGF-induced MIF transcription and direct bevacizumab interaction, because of the 31% protein homology between MIF and VEGF in the binding domain. Similar results have been also found with anti-VEGFR-2 therapy, presumably due to the impediment of MIF secretion [92]. A summary of the preclinical evidence of a therapeutic role for MIF signaling inhibition in GBM is presented in Figure 4.

## CONCLUSIONS

High grade gliomas still represent an unmet medical need, entailing poor prognosis and short life expectancy. Intensive research efforts have identified several etiopathogenetic mechanisms underlying GBM and various drugs are currently synthesized and developed as possible therapeutic strategies.

Taking advantages of the deeper information available on the MIF family in oncogenesis, and GBM in particular, additional *in vitro* and *in vivo* studies are warranted to determine the contribution of MIF and D-DT/MIF2 to the pathogenesis of GBM and the feasibility of their inhibitors as therapeutic approach. The recent identification of D-DT/MIF2 and its synergistic action in oncogenesis with MIF suggests that approaches able to simultaneously counteract both cytokines could be more effective than what it has been so far observed with standard single MIF inhibition. In this regard, the use of the firstly identified dual inhibitor, 4-IPP, might offer an accurate understanding of the advantages of developing this compound and other dual inhibitors over standard single MIF inhibitor. It will also be important to evaluate the possible synergistic action of MIF-inhibitors with standard of care treatment of GBM and new experimental approaches including immune check-point inhibitors that are being evaluated in Phase I/II studies in GBM [NCT02852655, NCT02617589, NCT02798406, NCT03277638]. Also, brain-targeted delivery of drugs using nanotechnology may help improve the success rate of current pharmacological interventions. A number of clinical trials are ongoing using liposomes but this area is still in its infancy. PNPs have already been used to deliver MDR-1 gene silencing siRNA and paclitaxel to chemotherapy-refractory ovarian adenocarcinoma cells [136] therefore, combination therapy using both gene silencing and conventional chemotherapeutics, as well as, small inhibitors, may represent in the future, a promising avenue.

Finally, identifying MIF family biomarkers suggestive of a response to MIF inhibitors including MIF and MIF2 blood levels and genetic polymorphisms of these cytokines and their receptors will also be necessary to accurately identify the eventual subset of GBM patients more likely to respond to MIF-DDT inhibition.
